# Hepatoprotective Effects of Zerumbone against Paracetamol-Induced Acute Hepatotoxicity in Rats

**DOI:** 10.21315/mjms2018.25.2.7

**Published:** 2018-04-27

**Authors:** Asmah Hamid, Liow Say Lee, Saiful Ridzuan Karim, Nurul Farhana Jufri

**Affiliations:** Biomedical Science Programme, Centre for Health & Applied Sciences, Faculty of Health Sciences, Universiti Kebangsaan Malaysia, Jalan Raja Muda Abdul Aziz, 50300 Kuala Lumpur, Malaysia

**Keywords:** zerumbone, paracetamol, oxidative stress, liver, toxicity

## Abstract

**Background:**

Zerumbone (ZER) is a major bioactive compound of *Zingiber zerumbet,* a wild ginger plant that has been documented to have anti-proliferative, anti-inflammatory and anti-oxidant properties. To investigate its hepatoprotective potential, this study was designed to determine the treatment effects of ZER on acute hepatotoxicity induced by paracetamol (PCM) in rats.

**Methods:**

The control group was administered with phosphate buffer solution (PBS) while the other two groups received PCM alone (1000 mg/kg) and PCM + 25 mg/kg ZER, respectively, at 0 h and 4 h after PCM injection. After 24 h, the blood and liver were collected for differential white blood cell count, liver histological observation and biochemical analysis including alanine aminotransferase (ALT), aspartate aminotransferase (AST), and total protein concentration in serum and liver.

**Results:**

Treatment with ZER was found to significantly reduce ALT (*P* = 0.041), AST (*P* = 0.044) and total hepatic protein (*P* = 0.045) in comparison to PCM-induced rats. Rats treated with ZER exhibited the normal structure of hepatocytes with no vacuolisation or necrosis and showed significantly reduced neutrophil count (*P* = 0.037). This finding suggests its ability to suppress the inflammatory processes caused by PCM overdosage and decrease the hepatocytes tendency to go through necrotic processes.

**Conclusion:**

ZER possessed protective activity against PCM-induced acute hepatotoxicity in a rat model.

## Introduction

Paracetamol (PCM) is an analgesic and anti-pyretic drug that is widely used to reduce mild pain and headache. The consumption of PCM in high dosage is toxic to the human liver, but it is safe to be used at its therapeutic dose as it will be biotransformed and eliminated as non-toxic conjugates of sulphate and glucuronic acid (1). PCM overdosage will lead to hepatic necrosis and lesion, kidney injury or even death to humans and experimental animals (2). A reactive metabolite of PCM, *N*-acetyl-para-benzo-quinone imine (NAPQI), is believed to cause the hepatic damage in PCM overdosage (3).

Nowadays, studies are extensively exploring natural products to maintain the liver function and treat diseases of the liver. Ginger is an example of a bioactive compound that is rapidly gaining popularity among the modern researchers. The rhizome of ginger is the medicinally useful part (4). *Zingiber zerumbet*, which is locally known as ‘lempoyang’, is wild ginger belonging to the Zingiberaceae family. Its rhizome is commonly used in traditional medicines to treat swelling, sore throat and loss of appetite (5). Meanwhile, the juice from boiled rhizome is used to treat jaundice in the local community (6).

Zerumbone (ZER) is a sesquiterpene isolated from the rhizomes of *Z. zerumbet* (7). It is a major active compound found in the rhizomes of this plant and has been reported to have an anti-tumour promoter, anti-proliferative, anti-inflammatory, and anti-oxidant properties (5, 8–10). Moreover, ZER has also been reported to exert its anti-microbial and anti-leukemic effects and activates phase II drug metabolising enzymes (11–13).

Recently, *Z. zerumbet* ethyl acetate extract has been documented to have hepatoprotective effects on PCM-induced hepatotoxicity in rats (14). However, the effects of ZER against PCM-induced hepatotoxicity have not been widely investigated. Consequently, the aim of this present study is to determine the treatment effects of ZER on PCM-induced hepatotoxicity in rats.

## Material and Methods

### Zerumbone

ZER (2,6,9,9-tetramethyl-[2*E*,6*E*,10*E*]-cycloundeca-2,6,10-trien-1-one) was obtained as a gift from the Department of Chemistry, Faculty of Science, Universiti Teknologi Malaysia, Skudai, Johor, Malaysia. Briefly, the rhizomes were chopped, dried at room temperature and subjected to hot extraction of Soxhlet apparatus for 18 h using *n*-hexane as the solvent. The solvent was evaporated using rotatory vapour to produce a crude extract, and it was recrystallised using hot *n*-hexane overnight to produce white crystalline solid, ZER (15).

### Animal

Twenty-four adult male Sprague-Dawley rats were obtained from the Animal Unit, Institute of Medical Research–Universiti Kebangsaan Malaysia (IMR–UKM), Kuala Lumpur. The animals’ weight ranged from 240 g–340 g. One week prior to the start of the study, the animals were housed under the standard condition and fed with standard pellet and water ad libitum. All experimental procedures were carried out with the approval of Animal Ethics Committee of UKM.

### Experimental Design

Rats were randomly assigned into three groups with each group consisting of six rats. The group I consisted of control rats who were given 1 mL phosphate buffer solution (PBS) at 0 h and 4 h. The group II rats received an overdose of PCM (1000 mg/kg) and 1 mL PBS at 0 h and 4 h. The group III rats received an overdose of PCM (1000 mg/kg) and ZER (25 mg/kg) at 0 h and 4 h. Both PCM and ZER were administered to the rats via intraperitoneum injection.

After 24 h of PCM treatment, the rats were anaesthetised with KTX (ketamine, zylazil, xoletil), and blood sample was taken via cardiac puncture. Blood samples were collected and processed for biochemical assays. Then the rats were sacrificed by cervical dislocation. The liver was removed and cleaned, and then small sections were thinly sliced before being preserved in 10% formalin solution for histological section. The remaining liver tissues were homogenised in 1.15% potassium chloride solution at 3 mL/kg (v/w) for biochemical analysis.

### Differential White Blood Count

A thin blood film was prepared from a drop of full blood taken by cardiac puncture on a clean glass slide. Dried blood slide was stained with Wright stain.

### Assessment of Liver Function

Blood was left to stand for 3 h in a centrifuge tube without anti-coagulant before the samples were centrifuged at 3000 rpm at 4 °C for 10 min to separate the serum. For the liver function test, liver aspartate aminotransferase (AST) and alanine aminotransferase (ALT) activities were evaluated using the method by Reitman and Frankel (16). Serum and liver total protein were analysed according to the method suggested by Bradford (17).

### Histopathological Studies

The liver tissues with approximately 5 mm thickness were fixed in 10% formalin solution for 48 h and dehydrated with increasing sequences of alcohol concentrations from 50% (2 h), 70% (overnight), 90% (2 h) and 100% (2 h and overnight). The samples were then cleared by xylene and embedded in paraffin. The samples were cut into 5 mm–7 mm thickness and stained with hematoxylin and eosin dye for microscopic observation of histopathological changes.

### Statistical Analysis

Data were analysed with one-way ANOVA with post-hoc Tukey test to assess the differences between groups by using SPSS version 20.0 software. All results are presented as mean [SEM], and an exact *P*-value below 0.050 will be considered as statistically significant.

## Results

In this study, the rats treated with PCM showed a significantly higher activity of liver function markers, ALT and AST enzymes as compared to the control group (Table 1). Administration of ZER at the dose of 25 mg/kg after induction of hepatotoxicity by PCM significantly lowered the level of serum AST (*P* = 0.044) and ALT (*P* = 0.041).

Evaluation of serum and hepatic total protein showed a significant increase in the concentration of protein in both serum and liver tissues from PCM-treated group (Group II) in comparison with the control group. As for group III, the concentration of total protein in liver tissue was significantly decreased after treatment with ZER (*P* = 0.045), whereas only a slight decrease was seen in serum total protein (Table 1).

Differential white blood cells count performed on thin blood films showed that Group II (PCM) had a lower lymphocyte count but an elevated level of neutrophil as compared to control group (*P* = 0.047). Treatment with ZER at 25 mg/kg in Group III reduced the level of neutrophil (*P* = 0.037) to near the normal level as compared to the control group. The eosinophil count was slightly decreased in Group II while the number was increased non-significantly in Group III. For basophil and monocyte, there were no significant difference between the three treatment groups (Table 2).

**Figure 1:**
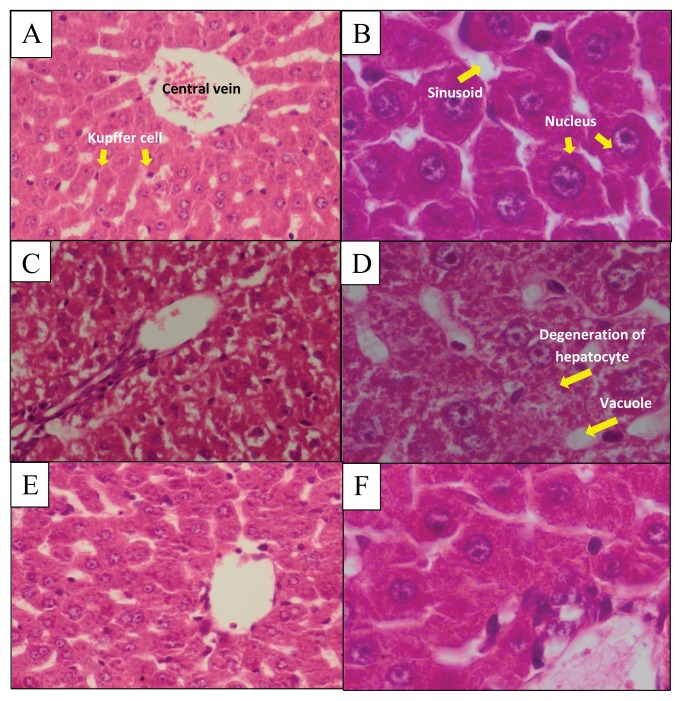
The effect of zerumbone on paracetamol-induced liver damage in rat. A and B show the liver from rats in control group with normal shaped hepatic cells uniformly arranged surround a central vein, sinusoid spaces, Kupffer cells and nucleus. C and D show the liver histology from rats treated with PCM only with necrosis and vacuolisation of hepatic cells with degeneration of hepatocytes. E and F show the liver histology from the group treated with PCM and ZER with normal shaped hepatocyte with no vacuolisation or necrosis. A, C and E 400× magnification; B, D and F 1000× magnification

Histopathological studies (Figure 1) showed that the control group exhibited normal cellular architecture of liver cells, which were uniformly arranged surrounding the central vein. The polyhedral shaped hepatocyte contained a nucleus of normal size. Sinusoidal space and Kupffer cells were also observed. Liver histology from Group II (PCM) showed the presence of centrilobular necrosis, vacuolisation and hepatocyte degeneration with the characteristics of pyknosis, karyorrhexis and karyolysis in the nucleus. Administration of ZER after the induction of hepatotoxicity by PCM (Group III) displayed a normal structure of the hepatic tissue.

## Discussion

Rats treated with PCM at 1000 mg/kg dosage showed a significantly elevated level of ALT and AST enzymes in biochemical analysis. The dose selection was based on a previous study by Hinson et al. (3) and, Price and Jollow (18) who demonstrated that PCM dosage at 1000 mg/kg intraperitoneally could cause liver necrosis in rats. Increased levels of both parameters indicate the hepatic damage caused by an overdose of PCM as reported in previous research, which demonstrated that rats treated with 1000 mg/kg of PCM developed hepatic necrosis (18). Liver injury will release the hepatic enzymes (ALT and AST) into the blood stream, and this will lead to an increased level of both enzymes in the blood samples (19).

Liver cell damage induced by overdosage of PCM is caused by the overproduction of PCM reactive toxic metabolite, NAPQI (4). Excessive reaction of NAPQI with mitochondrial protein caused oncotic necrosis in hepatocytes, which is also believed to play its role in the increased levels of ALT and AST (20). In addition, PCM-induced hepatic damage may be due to mitochondria permeability transition that contributed to mitochondrial oxidative stress and ATP depletion (21).

In this research, 25 mg/kg of ZER was administered to the rats in the test group in order to investigate its protective potential on PCM-induced toxicity in rats. Selection of the dosage is based on the previous research on curcumin by Kheradpezhouh et al. (22). It has been suggested that ZER exerts its hepatoprotective effect by stimulating or activating phase II drug metabolising enzymes (23). Activation of phase II xenobiotic metabolic enzymes (GST) suppresses the production of oxidative stress produced by the binding of NAPQI with hepatocytes.

In this present study, the administration of ZER after treatment with PCM showed a significantly lower value of AST and ALT as compared to the group treated with PCM only. These results are in accordance with the finding of Asmah et al. (14) who reported the hepatoprotective effect of *Z. zerumbet* ethyl acetate extract at the concentrations of 200 mg/kg and 400 mg/kg as demonstrated by a significant reduction in liver enzymes level in rats. ZER is one of the major active compounds found in the rhizome of *Z. zerumbet* and is believed to exert this protective action. These results also support the study conducted by Fakurazi et al. (24) that found the reduced levels of ALT and AST in PCM-induced hepatoxicity in rats after being treated with different concentrations of ZER.

The elevated level of hepatic and serum total protein after administration of 1000 mg/kg PCM might be due to the increased synthesis of acute phase protein (APP) in the liver during acute inflammation process (25). This occurred after depletion of glutathione storage in the liver as glucuronidation and sulphonation pathways became saturated, causing a majority of PCM to be metabolised to NAPQI by the CYP 2E1 pathway (4). Meanwhile, the decreased level of total protein in rats treated with PCM and ZER may indicate the anti-inflammatory effect of ZER, as suggested by Takada et al. (26) who reported that ZER inhibited inflammation process by suppressing the NF-κB transcription activity.

Lymphocyte and neutrophil are responsible for the phagocytosis of apoptotic bodies produced by hepatic PCM toxicity. The increased number of neutrophil after administration of PCM at 1000 mg/kg (group II) in this research is in accordance with the finding reported by Smith et al. (27). A previous study reported that rat hepatocytes exposed to PCM produced chemotactic factor, which increased the neutrophil chemotaxis (28). Interestingly, the administration of ZER at 0 and 4 h after administration of 1000 mg/kg PCM (group III) showed a significantly lower neutrophil count as compared to group II. The ability of ZER to activate phase II drug metabolising enzymes suggests that ZER may suppress the inflammation process caused by the overdosage of PCM and decrease the hepatocytes tendency to go through an apoptotic process (23). Consequently, phagocytosis are not induced in hepatocytes, thus lower the neutrophil count.

Results from the biochemical analysis were supported by histological investigation of hepatic tissue damage induced by PCM. This observation is in accordance with the previous findings that PCM caused centrilobular necrosis and degeneration of hepatocyte with the characteristic of pyknosis, karyorrhexis and karyolysis in the nucleus (29). The binding between NAPQI with hepatic macromolecules led to the disruption of calcium homeostasis in hepatic cells, leading to cell death (30). Meanwhile, the liver from the group treated with both PCM and ZER could maintain structural integrity as no hepatic vacuolisation or necrosis was observed. ZER at 25 mg/kg dosage is suggested to have a protective effect on hepatic cells, which may be caused by the anti-inflammatory properties of ZER and also its ability to activate phase II drug metabolising enzymes such as GST (23). Due to limited funding, the number of parameters that could be tested was limited. Another liver function test enzyme that can be measured is gamma-glutamyl transpeptidase. Future studies could investigate the protein profile after ZER administration to identify the proteins that are involved in the protective effect. In addition, we could explore the potential of ZER in protecting other liver-related diseases such as hepatocarcinoma or hepatitis.

## Conclusion

In conclusion, ZER has been shown to possess protective effects against PCM-induced acute hepatotoxicity in a rat model by the decreased level of AST, ALT and total protein in biochemical analysis. The normal structure of hepatocyte with no vacuolisation or necrosis and reduces lymphocyte and neutrophil count suggesting ZER ability to suppress the inflammatory processes.

## Figures and Tables

**Table 1:** Effect of zerumbone treatment on liver enzyme activity and total protein

Treatment	Mean (SEM)			

	Alanine Aminotransferase (ALT) (IU/L)	Aspartate Amiotransferase (AST) (IU/L)	Serum total protein (g/100 mL)	Hepatic total protein (g/100 mL)
Group I (Control)	30.18 (7.80)	40.07 (3.31)	41.46 (6.27)	28.68 (4.03)
Group II (PCM+PBS)	58.33 (4.17)^a^	66.69 (7.83)^a^	52.19 (6.84)^a^	45.10 (10.23)^a^
Group III (PCM + ZER 25 mg/kg)	35.63 (7.66)^b^	49.08 (4.34)^a^,^b^	47.24 (4.12)	21.29 (4.32)^a^,^b^

Values were expressed as mean (SEM)

a Significantly different from Group I (control group)

b Significantly different from Group II (group treated with PCM only)

**Table 2:** Differential white blood cell count of treated group

Treatment	Mean (SEM)				

	Basophil	Eosinophil	Lymphocyte	Monocyte	Neutrophil
Group I	Not detected	1.40 (1.52)	43.20 (6.14)	8.80 (3.96)	47.80 (8.82)
Group II	Not detected	0.50 (0.55)	23.67 (6.12)^a^	5.33 (4.18)	70.67 (6.31)^a^
Group III	Not detected	2.33 (3.14)	47.50 (9.35)^b^	6.17 (2.56)	44.00 (10.35)^b^

Values were expressed as mean (SEM)

a Significantly different from Group I (control group)

b Significantly different from Group II (group treated with PCM only)

## References

[1] Attia SM (2010). Deleterious effects of reactive metabolites. Oxid Med Cell Longev.

[2] Yoon E, Babar A, Choudhary M, Kutner M, Pyrsopoulos N (2016). Acetaminophen-induced hepatotoxicity: a comprehensive update. J Clin Transl Hepatol.

[3] Hinson JA, Roberts DW, James LP (2010). Mechanisms of acetaminophen-induced liver necrosis. Handb Exp Pharmacol.

[4] Ahmed RS, Suke SG, Seth V, Chakraborti A, Tripathi AK, Banerjee BD (2008). Protective effects of dietary ginger (*Zingiber officinales* Rosc.) on lindane induced oxidative stress in rats. Phytother Res.

[5] Somchit MN, Nur Syukriah MH (2003). Anti-inflammatory property of ethanol and water extract of Zingiber zerumbet. Indian J Pharmacol.

[6] Ong HC, Norzalina J (1999). Malay herbal medicine in Gemencheh, Negeri Sembilan, Malaysia. Fitoterapia.

[7] Murakami A, Takahashi M, Jiwajinda S, Koshimizu K, Ohigashi H (1999). Identification of zerumbone in *Zingiber zerumbet* Smith as a potent inhibitor of 12-O-tetradecanoylphorbol-13-acetate-induced Epstein-Barr virus activation. Biosci Biotechnol Biochem.

[8] Murakami A, Tanaka T, Lee JY (2004). Zerumbone, a sesquiterpene in subtropical ginger, suppresses skin tumor initiation and promotion stages in ICR mice. Int J Cancer.

[9] Kirana C, McIntosh GH, Record IR, Jones GP (2003). Anti-tumor activity of extract of *Zingiber aromaticum* and its bioactive sesquiterpenenoid zerumbone. Nutr Cancer.

[10] Ruslay S, Faridah A, Khozirah S, Zurina Z, Maulidiani, Hasnah S (2007). Characterization of the components present in the active fractions of health gingers (*Curcuma xanthorrhiza* and *Zingiber zerumbet*) by HPLC–DAD–ESIMS. Food Chemistry.

[11] Abdul A, Siddiq IA, Adel SA, Manal ME, Syam MM (2008). Anti-cancer and anti-microbial activities of zerumbone from the rhizomes of *Zingiber zerumbet*. Int J Pharm.

[12] Xian M, Ito K, Nakazato T (2007). Zerumbone, a bioactive sesquiterpene, induces G2/M cell cycle arrest and apoptosis in leukemia cell via a Fasand mitochondria-mediated pathway. Cancer Sci.

[13] Nakamura Y, Yoshida C, Murakami A, Ohigashi H, Osawa T, Uchida K (2004). Zerumbone, a tropical ginger sesquiterpene, activates phase II drug metabolizing enzymes. FEBS Letters.

[14] Asmah H, Siti BB, Rafidah APM, Pakri M, Noraida AM, Yuliani Y (2011). Role of oxidative stress in the protective effects of *Zingiber zerumbet* Smith ethyl acetate extract against paracetamol-induced hepatotoxicity in Sprague-Dawley rats. Australian Journal of Basic and Applied Sciences.

[15] Noor NFM, Sirat HM (2016). Isolation, characterization and modification of zerumbone from *Zingiber zerumbet*. eProceedings Chemistry.

[16] Reitman S, Frankel S (1957). Colorimetric method for the determination of serum transaminase activity. Am J Clin Pathol.

[17] Bradford MM (1976). A rapid and sensitive method for the quantitation of microgram quantities of protein utilizing the principle of protein-dye binding. Anal Biochem.

[18] Price VF, Jollow DJ (1986). Strain differences in susceptibility of normal and diabetic rats to acetaminophen hepatotoxicity. Biochem Pharmacol.

[19] Drotman RB, Lawhorn GT (1978). Serum enzymes as indicators of chemically induced liver damage. Drug Chem Toxicol.

[20] Jaeschke H, Bajt ML (2006). Intracellular signaling mechanisms of acetaminophen-induced liver cell death. Toxicol Sci.

[21] Masubuchi Y, Suda C, Horie T (2005). Involvement of mitochondrial permeability transition in acetaminophen-induced liver injury in mice. J Hepatol.

[22] Kheradpezhouh E, Panjehshahin MR, Miri R, Javidnia K, Noorafshan A, Monabati A (2010). Curcumin protects rats against acetaminophen-induced hepatorenal damages and shows synergistic activity with N-acetyl cysteine. Eur J Pharmcol.

[23] Nakamura Y, Yoshida C, Murakami A, Ohigashi H, Osawa T, Uchida K (2004). Zerumbone, a tropical ginger sesquiterpene, activates phase II drug metabolizing enzymes. FEBS Letters.

[24] Fakurazi S, Hairuszah I, Mohd Lip J, Shanti G, Nanthini U, Shamima AR (2009). Hepatoprotective action of zerumbone against paracetamol induced hepatotoxicity. J Med Sci.

[25] Jain S, Gautam V, Naseem S (2011). Acute-phase proteins: as diagnostic tool. J Pharm Bioallied Sci.

[26] Takada Y, Murakami A, Aggarwal BB (2005). Zerumbone abolishes NF-kappaB and IkappaBalpha kinase activation leading to suppression of antiapoptotic and metastatic gene expression, upregulation of apoptosis, and downregulation of invasion. Oncogene.

[27] Smith GS, Nadig DE, Kokoska ER, Solomon H, Tiniakos DG, Miller TA (1998). Role of netrophil in hepatotoxicity induced by oral acetaminophen administration in rats. J Surg Res.

[28] Takada H, Mawe E, Shiratori Y, Hikiba Y, Nakata R, Yoshida H (1995). Chemotactic factors released from hepatocytes exposed to acetaminophen. Dig Dis Sci.

[29] Toklu HZ, Sehirli AO, Velioglu-Ogunc A, Cetinel S, Sener G (2006). Acetaminophen-induced toxicity is prevented by β-d-glucan treatment in mice. Eur J Pharmacol.

[30] Sturgill MG, Lambert GH (1997). Xenobiotic induced hepatotoxicity: mechanisms of liver injury and methods of monitoring hepatic function. Clin Chem.

